# Autofluorescence-spectral imaging as an innovative method for rapid, non-destructive and reliable assessing of soybean seed quality

**DOI:** 10.1038/s41598-021-97223-5

**Published:** 2021-09-08

**Authors:** Clíssia Barboza da Silva, Nielsen Moreira Oliveira, Marcia Eugenia Amaral de Carvalho, André Dantas de Medeiros, Marina de Lima Nogueira, André Rodrigues dos Reis

**Affiliations:** 1 Center for Nuclear Energy in Agriculture (CENA), University of São Paulo (USP), Piracicaba, SP 13416-000 Brazil; 2 Department of Crop Science, College of Agriculture Luiz de Queiroz (ESALQ), University of São Paulo (USP), Piracicaba, SP 13418-900 Brazil; 3 Department of Genetics, College of Agriculture Luiz de Queiroz (ESALQ), University of São Paulo (USP), Piracicaba, SP 13418-900 Brazil; 4 Department of Agronomy, Federal University of Viçosa (UFV), Viçosa, MG 36570-900 Brazil; 5 Department of Biosystems Engineering, School of Sciences and Engineering, São Paulo State University (UNESP), Tupã, SP 17602-496 Brazil

**Keywords:** Drug discovery, Biochemistry, Enzymes, Structural biology, Infectious diseases, Respiratory tract diseases

## Abstract

In the agricultural industry, advances in optical imaging technologies based on rapid and non-destructive approaches have contributed to increase food production for the growing population. The present study employed autofluorescence-spectral imaging and machine learning algorithms to develop distinct models for classification of soybean seeds differing in physiological quality after artificial aging. Autofluorescence signals from the 365/400 nm excitation-emission combination (that exhibited a perfect correlation with the total phenols in the embryo) were efficiently able to segregate treatments. Furthermore, it was also possible to demonstrate a strong correlation between autofluorescence-spectral data and several quality indicators, such as early germination and seed tolerance to stressful conditions. The machine learning models developed based on artificial neural network, support vector machine or linear discriminant analysis showed high performance (0.99 accuracy) for classifying seeds with different quality levels. Taken together, our study shows that the physiological potential of soybean seeds is reduced accompanied by changes in the concentration and, probably in the structure of autofluorescent compounds. In addition, altering the autofluorescent properties in seeds impact the photosynthesis apparatus in seedlings. From the practical point of view, autofluorescence-based imaging can be used to check modifications in the optical properties of soybean seed tissues and to consistently discriminate high-and low-vigor seeds.

## Introduction

Seeds are important sources of food for human and livestock, in addition to be considered the best natural method for protection of genetic material variability. Soybean [*Glycine max* (L.) Merril] is one of the most important oil and protein crop worldwide. The soybean yield depends on the use of high-vigor seeds because they guarantee a rapid and uniform establishment of plants under a variety of environmental conditions^1–3^. Nowadays, the seed industry uses standardized germination and vigor tests to predict field performance of seedlots. These tests provide valuable information on seed physiological potential; however, they are relatively time-consuming with subjective results that depend on specialized analysts, being difficult to reproduce the results^4^. Hence, there is a great interest for exploring advanced and innovative methods to further refine the current seed testing methods.

In the last few years, fluorescence-based methods are providing significant advances in many areas of life science, including medical diagnostics^5^, forensics^6^, food quality^7,8^, plant physiology^9^ and genetic analysis^10^. Traditional fluorescence imaging is mainly based on fluorescence spectroscopy (e.g. FTIR or Raman microspectroscopy) that can be applied to determine autofluorescent compounds, or non-fluorescent compounds can be measured by using multiple fluorescent tracers to highlight molecular, physiological or anatomical features^11,12^. However, this technique assesses only a small part of an object (i.e., a “spot measurement”), so they do not provide spatial information that it is important for many seed inspection applications. In this context, advanced autofluorescence-spectral imaging techniques can be a great alternative to overcome such a limitation. Autofluorescence-spectral imaging is a modern optical technology that combine both spatial and spectral information in a single measuring protocol. It provides a high-resolution optical spectrum for each image pixel and yields a set of images of the same object, in which each image is represented by a specific wavelength. In seed technology, autofluorescence-spectral imaging technique can be applied on fluorescent chemical compounds—the so-called fluorophores—with important roles in seed biology, including pigments (e.g. chlorophylls) and structural components of cell wall such as lignin^13,14^.

When fluorescent compounds are stimulated by light, they are raised to an excited state as a result of photon absorption, and the radiation light re-emitted is measured by autofluorescence-spectral imaging sensors. Photon absorption and fluorescence emission occur simultaneously^15^. Excitation is induced in the ultraviolet and visible regions, and a charge-coupled device chip (CCD-chip) combined with different filters at longer wavelengths detect the light emission (fluorescence) as seed compounds relax to lower energy levels. Furthermore, combining autofluorescence-spectral imaging technology with chemometric approaches may allow better characterization of seed quality in a non-destructive way. For instance, machine learning algorithms such as artificial neural network (ANN), support vector machine with either linear (SVM-*l*) or radial basis (SVM-*r*) kernel, and linear discriminant analysis (LDA) have been proven to be effective for solving problems in many research fields^16–20^. In the present study, we tested the use of these machine learning algorithms combined with autofluorescence-spectral imaging for classification of soybean seeds based on their physiological quality levels.

## Results

### Physiological status of seeds

**Figure 1:**
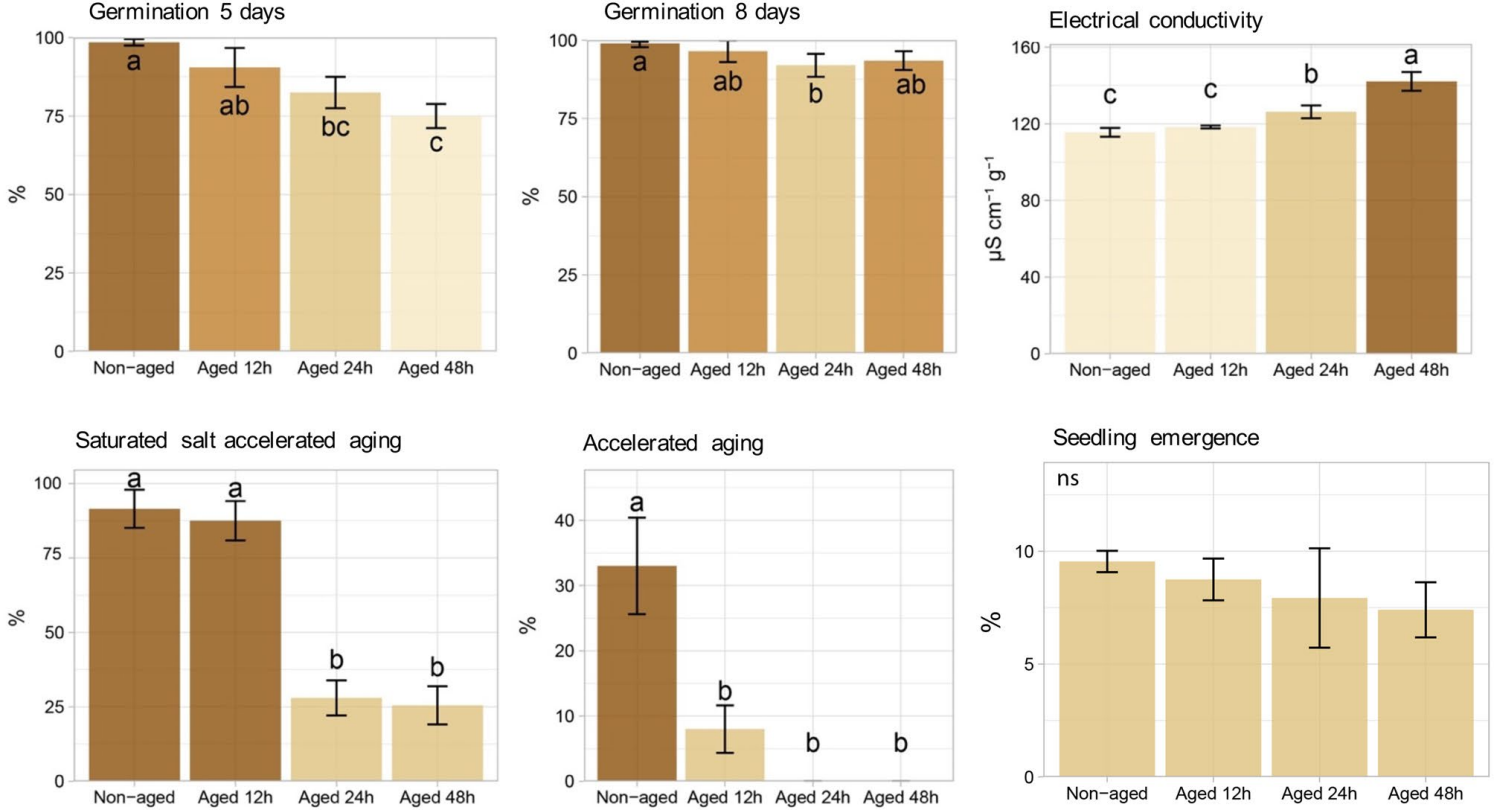
Germination and vigor tests for classes of non-aged soybean seeds and seeds aged for 12, 24 and 48 h. Means (± standard deviation) followed by the same letter are not significantly different according to Tukey test (*P* < 0.05).

The germination percentage decreased as seed aged, especially at 5 days after sowing (Fig. 1). While the electrical conductivity (an indirect indicator of seed membrane integrity) increased in seeds aged for 24 h and 48 h (Fig. 1), the saturated-salt accelerated aging (SSAA) separated seeds into two groups, i.e., seeds aged for 0 and 12 h *vs* seeds aged for 24 and 48 h. Meanwhile, the traditional artificial aging (AA) further sliced non-aged seeds from the aged ones. The seedling emergence test was unable to differ seed classes (Fig. 1).

### Biochemical changes in seeds

**Figure 2:**
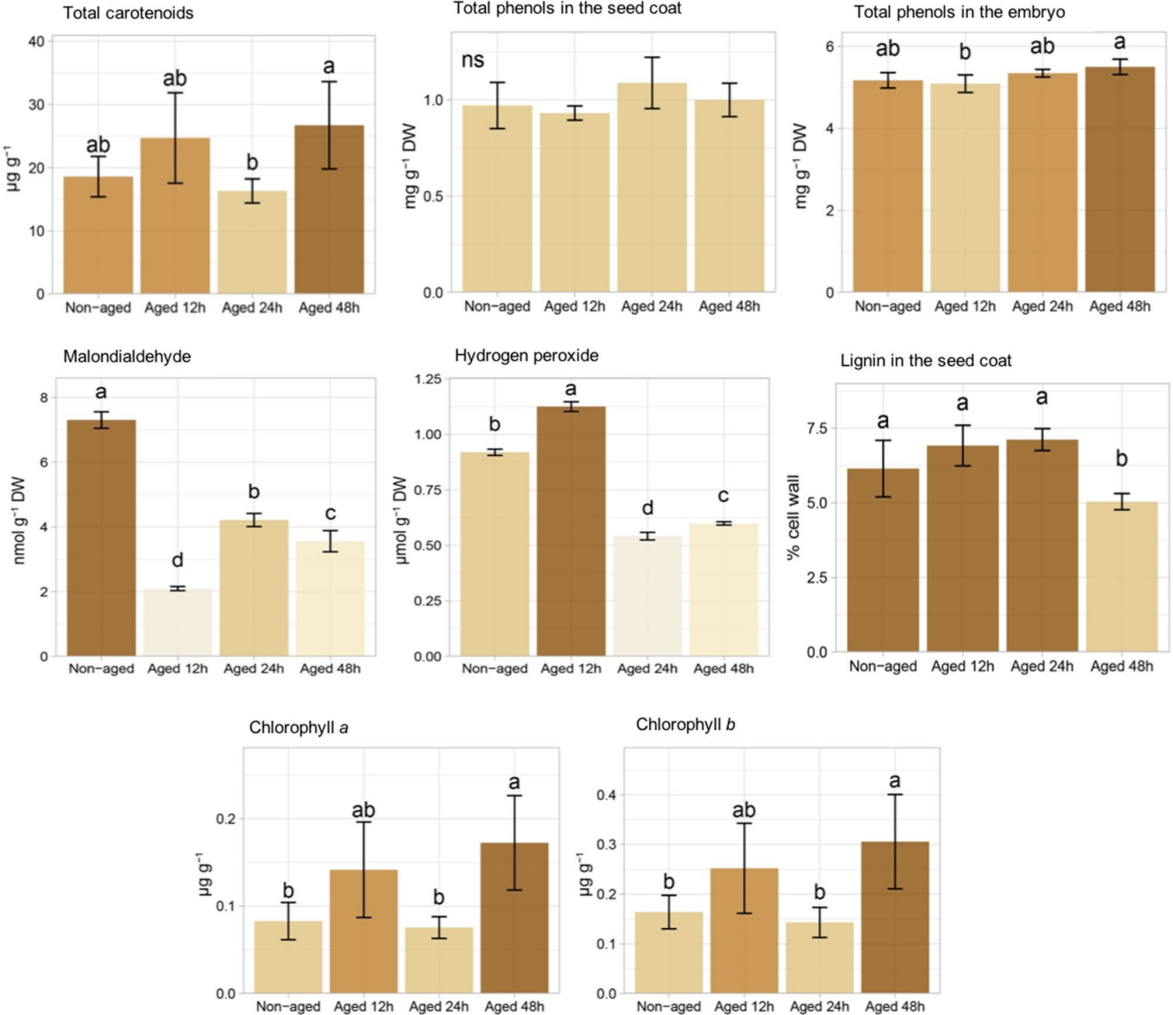
Total carotenoids, total phenols in the seed coat and embryo, malondialdehyde (MDA), hydrogen peroxide (H_2_O_2_), lignin in the seed coat, chlorophyll *a* and *b* of non-aged soybean seeds and seeds aged for 12, 24 and 48 h. Means (± standard deviation) followed by the same letter are not significantly different according to Tukey test (*P* < 0.05).

The total carotenoids and phenols in the seed coat and embryo (embryonic axis plus cotyledons) did not differ in aged seeds compared to non-aged class (Fig. 2). The malonaldehyde (MDA) content was markedly decreased in aged seeds in comparison to non-aged seeds (42.33–71.33%) (Fig. 2). In addition, there was a high amount of hydrogen peroxide (H_2_O_2_) in non-aged seeds, and a maximum level in seeds aged for 12 h (Fig. 2). Further biochemical analyses indicated that the content of lignin, chlorophyll *a* and *b* (Fig. 2) remained at similar levels for 0 (non-aged seeds), 12 and 24 h of aging; however, increasing the age time to 48 h reduced lignin content (by 14.23% compared to non-aged seeds). At the same time, seeds aged for 48 h had a substantial increment in chlorophyll *a* and *b* (108.09% and 86.50%, respectively).

### Autofluorescence-spectral markers

**Figure 3:**
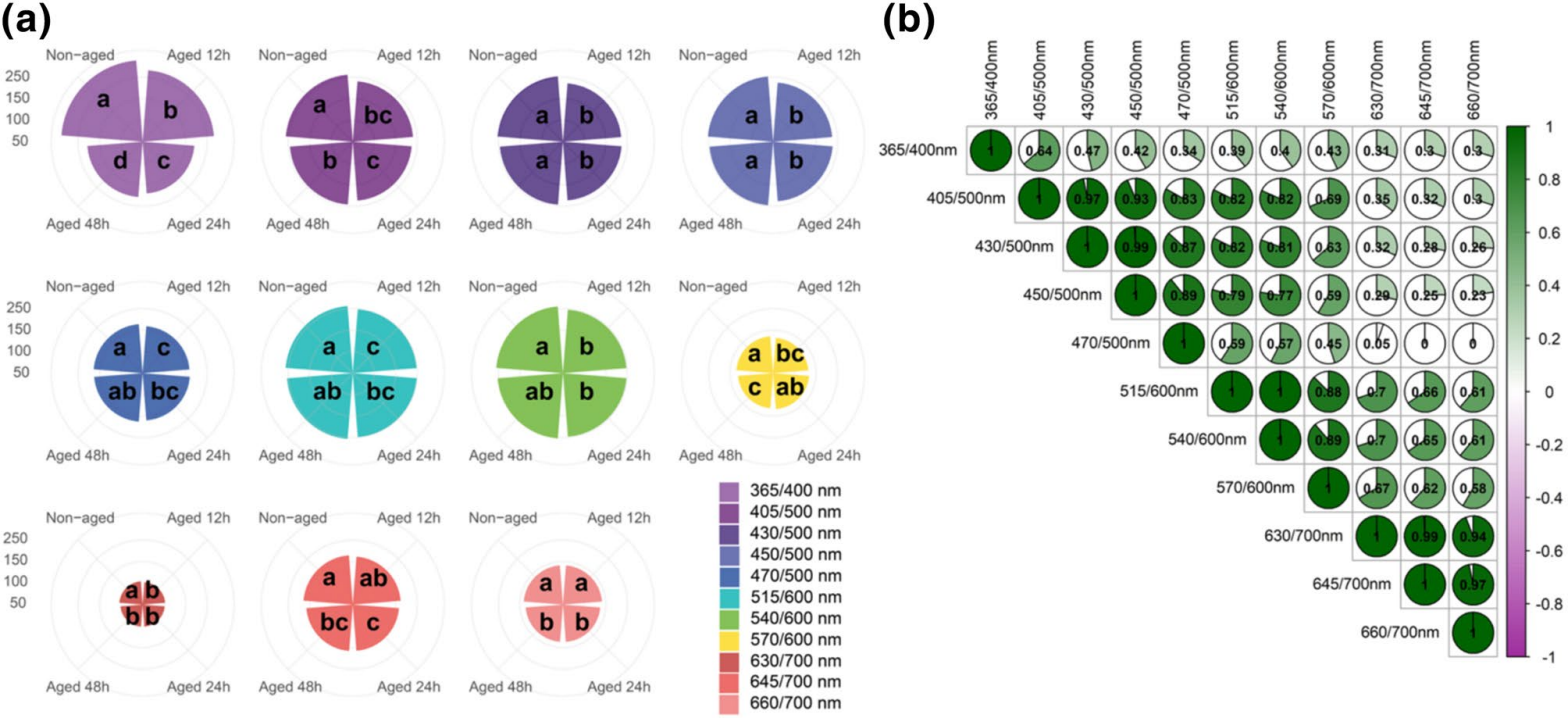
Comparison between classes of non-aged soybean seeds and seeds aged for 12, 24 and 48 h using autofluorescence-spectral data from different excitation-emission combinations; means followed by the same letter are not significantly different according to Tukey test (*P* < 0.05) **(a)**. Pearson’s correlation coefficients between all bandwidths used for autofluorescence-spectral imaging of soybean seeds (*n* = 200 seeds) **(b)**.

The average autofluorescence spectra of the seeds were higher at excitation wavelengths from 365 to 540 nm (with exception of 470 nm) (Fig. 3a). Non-aged seeds were efficiently separated from aged classes at 365/400 nm, 405/500 nm and 630/700 nm excitation-emission combinations; among these combinations, 365/400 nm provided the most refined discrimination of the seed classes (Fig. 3a). The highest correlation coefficients between bandwidths were: 1.00 (515/600 nm *vs* 540/600 nm), 0.99 (430/500 nm *vs* 450/500 nm; 630/700 nm *vs* 645/700 nm), and 0.97 (405/500 nm *vs* 430/500; 645/700 nm *vs* 660/700 nm) (Fig. 3b).

**Figure 4:**
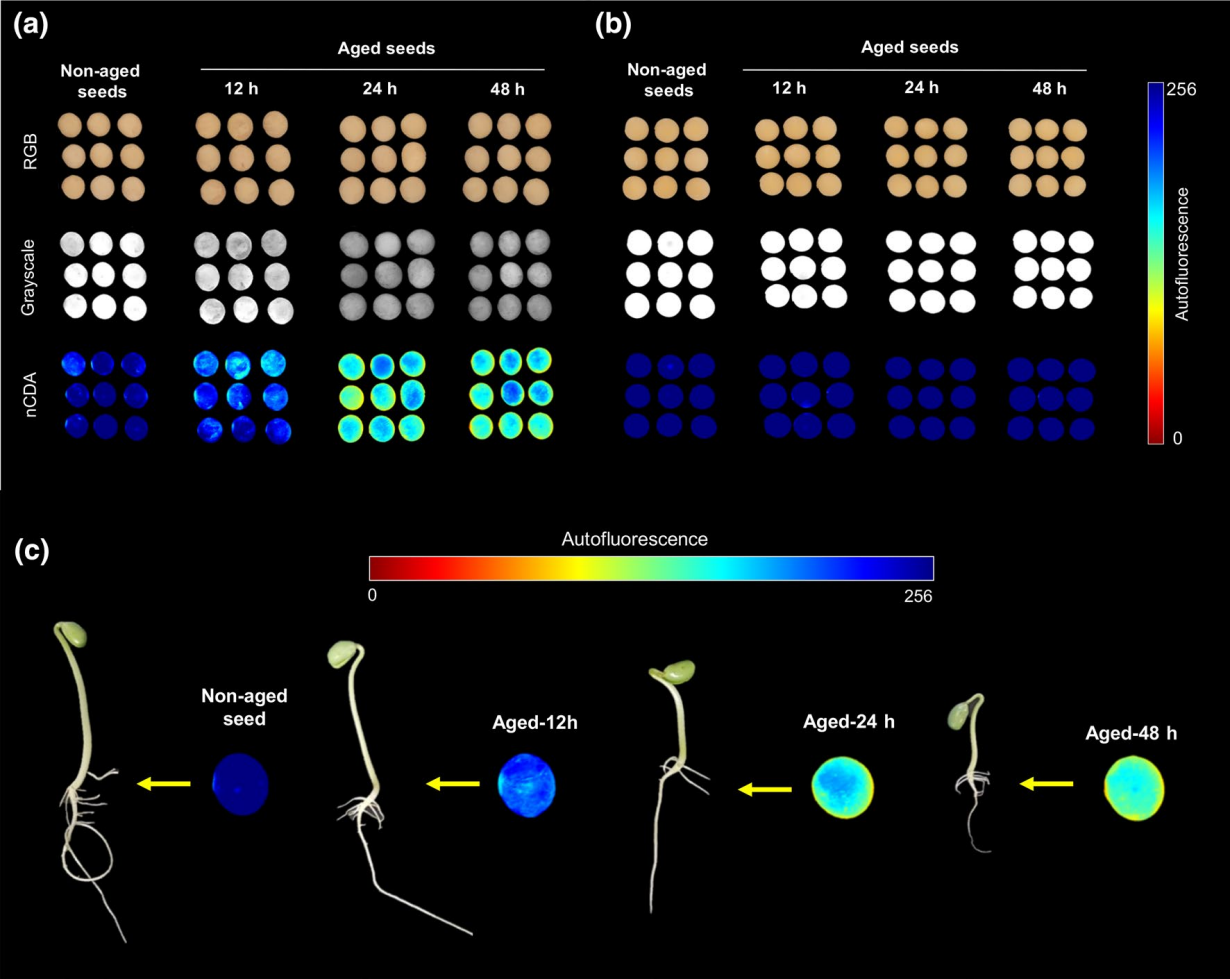
Raw RGB images from soybean seeds for classes of non-aged seeds and seeds aged for 12, 24 and 48 h, and corresponding autofluorescence images (grayscale and nCDA) captured at 365/400 nm excitation-emission combination, showing autofluorescence patterns in the presence **(a)** and absence **(b)** of the seed coat. Germination test at 8 days after sowing using seeds with different autofluorescence patterns in the nCDA images **(c)**. In the nCDA images, the pixel values (autofluorescence intensity) is calculated based on 10% trimmed mean to provide a more realistic image.

Using nCDA algorithm, the pixel values of the autofluorescence images acquired with the combination of 365/400 nm were transformed by removing the outlier observations, which allowed to calculate the trimmed mean. This technique is useful to eliminate the influence of outliers that can affect the mean, and thus providing a more realistic image. The autofluorescence intensity was displayed in the images using pixel-to-pixel mapping that was created using false color codes ranging from red (0) to blue (256), where higher pixel values represent tissues with higher autofluorescence (Fig. 4). Although all classes showed similar RGB images, there was a different autofluorescent pattern when the images were captured in the presence of the seed coat (Fig. 4a), in which seeds aged for 24 and 48 h exhibited the lowest autofluorescence signals (lower pixel values). However, removing the seed coat, all seed classes presented a similar autofluorescent pattern, regardless of the aging period (Fig. 4b). In the germination test at 8 days, the lower the seed autofluorescence signal in the presence of the seed coat the lower the seedling performance (Fig. 4c).

**Figure 5:**
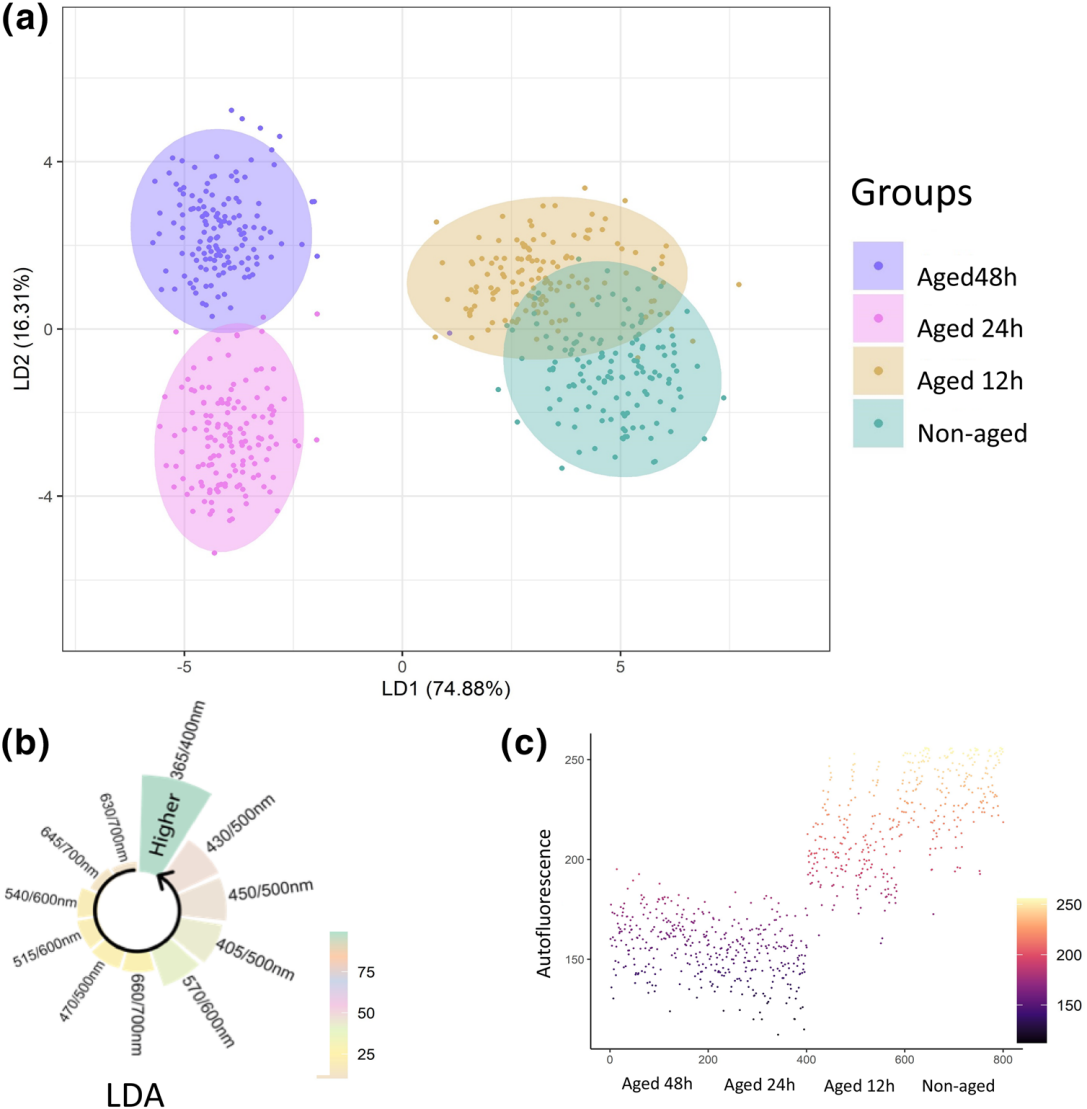
Linear discriminant analysis (LDA) for autofluorescence-spectral data in soybean seeds using different excitation-emission combinations **(a)**. Importance of each excitation-emission combination to discriminate non-aged seeds from seeds aged for 12 h, 24 h and 48 h **(b)**. Scatter plot with the representation of the values of each variable based on autofluorescence data using 365/400 nm combination **(c)** (*n* = 200 seeds).

In the linear discriminant analysis (LDA) the first two discriminant components (LD1 and LD2) explained 91.19% of the total variation between autofluorescence-spectral data (Fig. 5a). Autofluorescence data obtained from 365/400 nm combination contributed most to the LDA model (Fig. 5b), in which higher vigor seeds (i.e., non-aged seeds and seeds aged for 12 h) showed the highest fluorescence values (Fig. 5c).

### Seed quality classification based on different machine learning algorithms

The algorithms (i.e., ANN, SVM-*l*, and LDA) presented excellent performance for discrimination of seeds from different aged groups based on autofluorescence-spectral data (Table 1). In addition, the models developed showed very high accuracy (0.99), kappa (≥ 0.98), precision (0.99), recall (0.99), and F1 (0.99) in both cross and external validations (Table 1). The ANN algorithm showed slightly superior performance for kappa coefficient in cross-validation test.

**Table 1:** Comparation of the artificial neural network, support vector machine and linear discriminant analysis algorithms to assess soybean seed quality using autofluorescence-spectral data, and the model performances based on accuracy, kappa, precision, recall and F1.

Metrics	Artificial neural network		Support vector machine		Linear discriminant analysis	
	Cross-validation	External validation	Cross-validation	External validation	Cross-validation	External validation
	Folds = 5 (n = 680)	n = 120	Folds = 5 (n = 680)	n = 120	Folds = 5 (n = 680)	n = 120
Accuracy	0.99 ± 0.003	0.99	0.99 ± 0.010	0.99	0.99 ± 0.006	0.99
Kappa	0.99 ± 0.004	0.99	0.98 ± 0.013	0.99	0.98 ± 0.007	0.99
Precision	0.99 ± 0.003	0.99	0.99 ± 0.010	0.99	0.99 ± 0.005	0.99
Recall	0.99 ± 0.003	0.99	0.99 ± 0.010	0.99	0.99 ± 0.006	0.99
F1	0.99 ± 0.003	0.99	0.99 ± 0.010	0.99	0.99 ± 0.005	0.99

The models were created using autofluorescence-spectral data extracted from non-aged seeds and seeds aged for 12, 24 and 48 h (*n* = 800 seeds).

### Correlation between autofluorescence measurements and seed quality indicators

**Figure 6:**
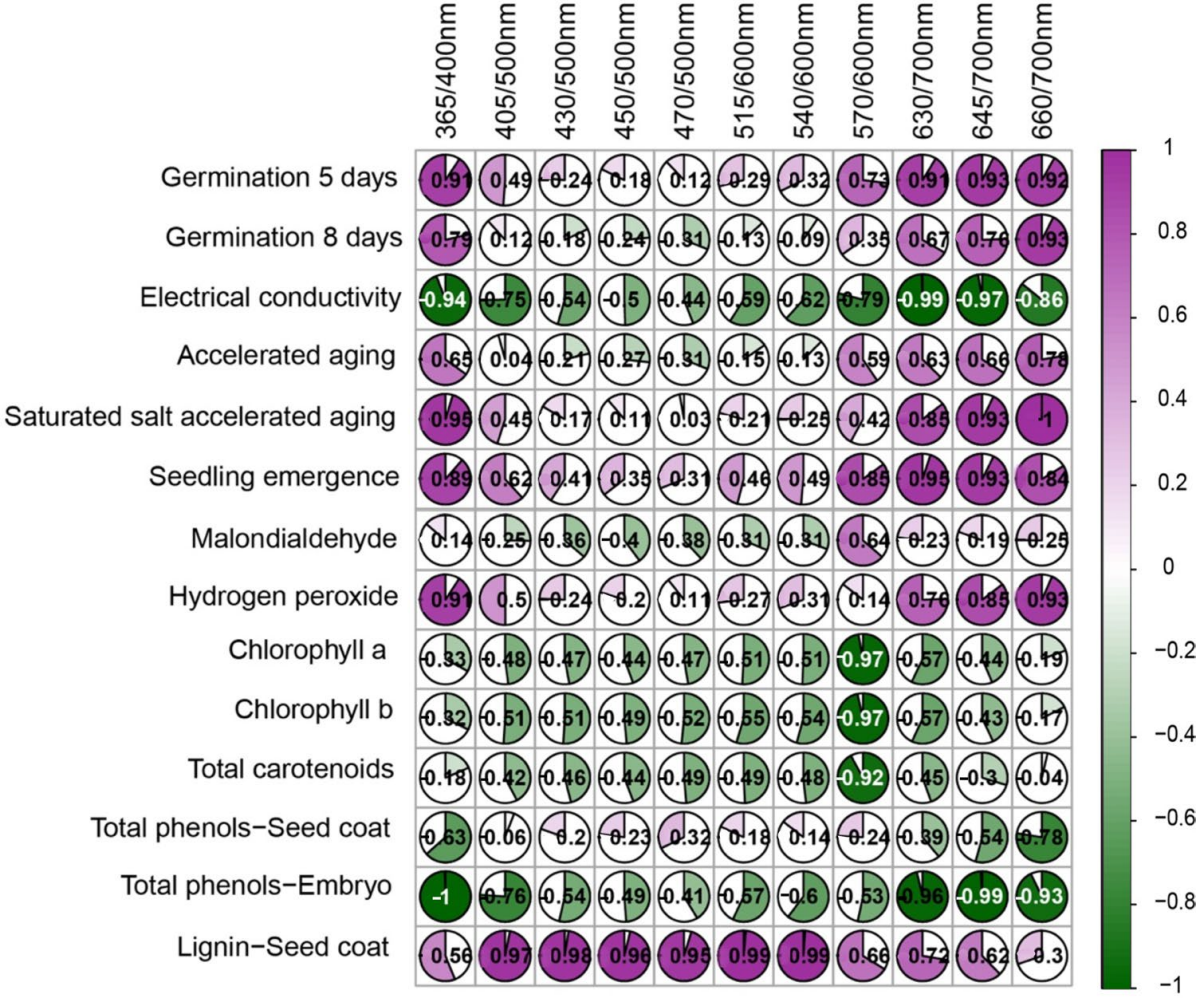
Pearson’s correlation coefficients between autofluorescence-spectral data from different excitation-emission combinations and soybean seed quality indicators.

The Pearson’s correlation analysis (Fig. 6) showed a perfect negative correlation between 365/400 nm combination and the total phenols in the embryo (− 1.00), and a perfect positive correlation between 660/700 nm combination and the SSAA test (1.00). High correlation coefficients were also noticed for other bandwidths: 0.99 (either 515/600 nm or 540/600 nm *vs* lignin), − 0.99 (630/700 nm *vs* electrical conductivity; 645/700 nm *vs* total phenols in the embryo), 0.98 (430/500 nm *vs* lignin), 0.97 (405/500 nm *vs* lignin), − 0.97 (570/600 nm *vs* either chlorophyll *a* or chlorophyll *b*), 0.96 (450/500 nm *vs* lignin) and 0.95 (470/500 nm *vs* lignin).

### Seedings grown from aged and non-aged seeds

**Figure 7:**
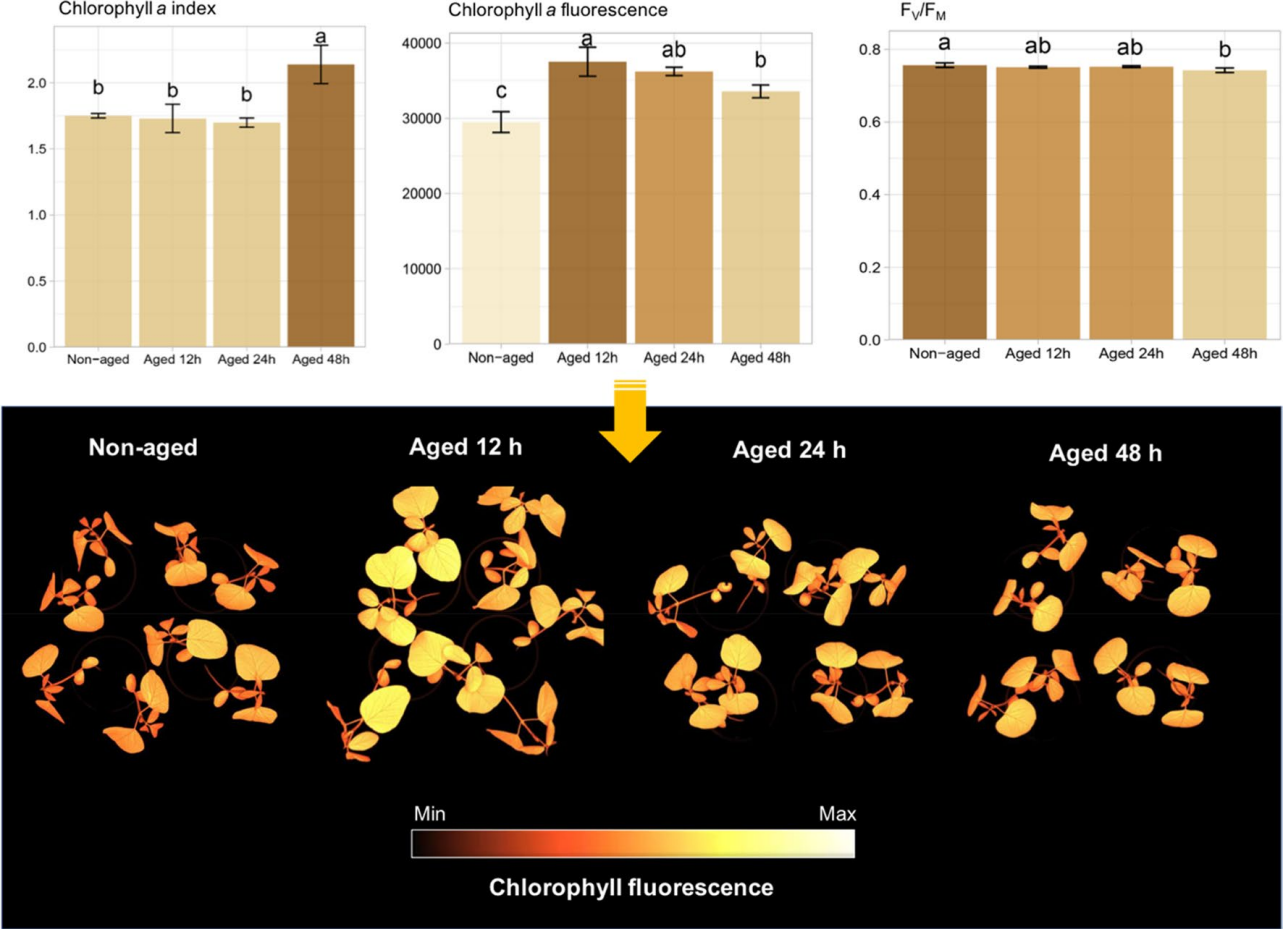
Chlorophyll *a* index, chlorophyll *a* fluorescence, and maximum quantum yield of photosystem II based on F_V_/F_M_ in soybean seedlings grown from non-aged seeds and seeds aged for 12, 24 and 48 h on the 16th day after sowing. Means (± standard deviation) followed by the same letter are not significantly different according to Tukey test (*P* < 0.05).

Although seedlings from seeds aged for 48 h reached a germination percentage similar to non-aged seeds at 8 days after sowing (Fig. 1), with a remarkably enhanced chlorophyll *a* index in the resulting seedlings on the 16th day (Fig. 7), they exhibited less efficient photochemical apparatus than those from non-aged seeds as highlighted by the quantum yield of photosystem II (F_V_/F_M_) (Fig. 7). Higher chlorophyll fluorescence was closely related to aged class, particularly in seedlings grown from seeds aged for very short period (12 h) (Fig. 7).

## Discussion

The vigor of seeds depends essentially on their ability to withstand the deleterious effects of aging, which induces the deterioration of tissues through consumption of reserves and damages to genetic material^21^. Artificial seed aging has been widely used to study the physiological and biochemical mechanisms associated with decreased seed vigor^22–24^, particularly to predict the seed performance during the entire storage period. Seed storage starts in the field when the seeds reach physiological maturity and ends at the beginning of the germination process^25^. For instance, at the point of physiological maturity, soybean seeds have a relatively high water content, and therefore seed quality can be highly affected by environmental conditions until harvest maturity, i.e., when the seeds reach a moisture content level that permits an efficient harvest. In the present study, we used an advanced autofluorescence-spectral imaging and machine learning models to efficiently classify the physiological potential of soybean seeds.

The artificial aging promoted deterioration to the soybean seed tissues as revealed by reduced physiological performance (Fig. 1) and biochemical changes (Fig. 2). For instance, lower seed germination was accompanied by loss of membrane integrity over time (as shown by progressive increase in electrical conductivity through the electrolyte leakage—Fig. 1). Another interesting result was the strong accumulation of H_2_O_2_ in the first aging period (12 h) (Fig. 2). Despite the fact that H_2_O_2_ can be a toxic byproduct of aerobic metabolism, depending on the concentration its role as a signaling molecule during oxidative stress is well-known^26^. Perhaps, as a consequence of H_2_O_2_ signaling, there was less damages to membrane polyunsaturated fatty acids^27^ with decreased MDA levels (a toxic aldehydic end product of lipid peroxidation of polyunsaturated fatty acids) during aging, as shown in Fig. 2. In this context, H_2_O_2_ content can be used as an indicative of “metabolic health” in soybean seeds. Accordingly, autofluorescence signals emitted in the excitation-emission combinations of 365/400 nm and 660/700 nm showed a very strong positive correlation with H_2_O_2_ levels (*r*^2^ = 0.91 and 0.93, respectively), which were two of the four bandwidth combinations strongly correlated with early germination test (*r*^2^ = 0.91 and 0.92, respectively). In addition, the combination of 365/400 nm was perfectly correlated with the accumulation of the total phenols in the seed embryo (*r*^2^ =  − 1.00), which efficiently protect soybean seeds from the oxidative stress of various origins ^28^. Meanwhile, the combination of 660/700 nm reached a perfect positive correlation with seed tolerance to the stressful conditions of the SSAA test (*r*^2^ = 1.00).

The nature of the autofluorescence intensity at excitation wavelength of 365 and 660 nm is mainly attributed to chlorophylls^29,30^, which absorb light in a broader range of the spectrum. Therefore, chlorophyll fluorescence appeared to be an important indicator of soybean seed quality for agricultural industry; however, besides chlorophyll, other autofluorescent molecules can also be excited at 365 nm such as lignin and ferulic acid (weaker signal) present in the cell wall^29,31^. Curiously, the combination of 365/400 nm provided a better separation of the seed classes, in which the autofluorescence decreased as the aging time increased (Figs. 3, 4, 5). It is important to emphasize that such discrimination was more efficient than the early germination test, for example (Fig. 1).

Additionally, our results indicated that depending on the seed region and the aging period, the aging process can (i) enhances chlorophyll synthesis (Fig. 2), (ii) increases chlorophyll degradation (Fig. 4) or (iii) induces these two events in seeds. As shown in the autofluorescence images (Fig. 4), the deleterious effects of deterioration predominantly affected the autofluorescent compounds present in the superficial regions of the seeds. Donaldson and Williams^31^ have demonstrated that chlorophyll fluorescence is highly reduced in deteriorated tissues due to the loss of chlorophylls, whereas these regions generally show a substantial increase in green fluorescence associated with browning. In recent studies, Medeiros et al.^16^ investigated soybean seed images obtained in the visible spectrum, and these authors also found a strong correlation between seed coat patterns and seed vigor levels.

In the current study, the greatest autofluorescence-spectral signals shown in non-aged soybean seeds corroborates previous studies of Li et al.^32^ who verified that the average spectral fluorescence of viable soybean seeds (by 365 nm excitation) was higher in relation to non-viable seeds. Moreover, we developed models based on ANN, SVM-*l* and LDA algorithms and full excitation-emission combinations, and the models presented an excellent performance for soybean seed quality classification (0.99 accuracy). The ANN method was implemented with the multilayer perceptron (MLP) algorithm that trains using Backpropagation. Neural networks have been successfully applied to identify contaminants in seeds through image analysis and the topology of the multilayer perceptron (MLP) also proved to be the best in recognizing classes of triticale seeds^33^. The SVM-*l* algorithm showed to be efficient in discriminating high-and low-vigor soybean seeds compared to LDA models^34^. However, the LDA approach has achieved the highest training speed, with high potential to classify the quality of beans^35^ and oat^19^ seeds from multispectral sources.

Other interesting results were: (1) a direct relationship between very low autofluorescence in seeds at 365/400 nm combination (aging for 48 h—Fig. 3a) and lower F_V_/F_M_ in the resulting seedlings (Fig. 7); (2) increased synthesis of chlorophyll *a* and chlorophyll *b* in seeds (aging for 48 h—Fig. 2) and greater accumulation of chlorophyll *a* in seedlings (Fig. 7). Considering that the percentage of emerged seedlings was significantly similar between non-aged and aged seeds (Fig. 1), our data suggest a possible compensatory mechanism during seedling development to outstand aging side effects that were detected at early stage of germination. Such mechanism may be related to an increase in chlorophyll *a* and *b* in aged seeds and higher amount of chlorophyll *a* in the resulting seedlings to improve the conversion of light to chemical energy—although this was not sufficient to improve the photochemical activity of the photosystem II as revealed by F_V_/F_M_ values. In fact, chlorophyll plays an essential role in the physiological performance of mature seeds^36,37^, and plant responses to abiotic stress conditions^38^.

Taken together, seed aging impacted photon-emitting substances that were detected by autofluorescence-spectral imaging, which can be used as a new marker for qualitative analysis of soybean seeds. Soybean seed is very sensitive to environmental stress, leading to both nutritious and economic losses. Moreover, in a seed bank context this approach may improve the accuracy of quality tests, providing novel insights into the storage and use of soybean seed sources.

## Conclusions

Autofluorescence-spectral imaging based on fluorescent properties of seed tissues can be a potential tool for characterization of soybean seed physiological potential. This approach can rapidly assess soybean seed quality using artificial-aging treatment by applying artificial neural network, support vector machine or linear discriminant analysis, with an excellent performance (0.99 accuracy). The autofluorescence emitted in the excitation-emission combination of 365/400 nm (that exhibited a perfect correlation with the total phenols in the embryo) enables a clear separation of seed classes.

Furthermore, there is a strong correlation between autofluorescence-spectral data and several quality indicators, including early germination and seed tolerance to stressful conditions. In addition, altering the autofluorescent compounds in seeds impact the photosynthesis apparatus in seedlings. To further advance development, we strongly support in depth-studies with a larger number of crop cultivars to detect seed quality traits and evaluate differences among different genotypes.

We would like to emphasize that the artificial-aging method used in the present work was previously used by other authors, with excellent results in the development of new seed quality methods based on non-destructive techniques^39–41^. However, another important future study will be to compare the results obtained from artificially-aged seeds, as done in the current work, with those of naturally-aged seeds (see Rajjou et al.^42^). Indeed, it is known that the physiology of seed deterioration during storage is largely influenced by the physical state of the cytoplasm^43^, which depends on equilibrium RH during storage. Under dry conditions (as in commercial and genebank storage) this cytoplasm is glassy with very low molecular mobility, while under high humidity aging conditions (as employed in the current study) the cytoplasm is liquid with high molecular mobility. Despite these limitations we anticipate that autofluorescence-spectral imaging based on fluorescent properties of seed tissues will provide a very potent and convenient tool for characterization of soybean seed physiological quality.

## Materials and methods

### Plant material

The soybean seeds (M5705 IPRO cultivar) used in this study were kindly provided by Dr. Raquel Márcia Modena Wutzki, Seed Quality Manager at the Lagoa Bonita Company, in Itaberá city, São Paulo State, Brazil. Seeds were produced in the Fazenda Redomona (field 194S18), Arapoti city, Paraná State, Brazil during 2018/2019 crop season. All required approvals were obtained for the study, which complied with all relevant regulations. Seeds with an initial moisture content of 7.9% were artificially aged for 0, 12, 24 and 48 h. For aging, seeds were distributed over a single layer on a wire mesh screen suspended inside a covered box (11.0 cm × 11.0 cm × 3.5 cm) containing 40 mL of distilled water at 42 °C and 98 ± 2% relative humidity (RH). Subsequently, seeds were dried at 20 °C for 24 h to bring them back to their initial pre-aged moisture content before measurements.

### Autofluorescence-spectral imaging

Multispectral fluorescence images were captured from seeds (four 9-cm glass Petri dishes with 50 seeds per class) using a VideometerLab4™ device (Videometer A/S, Herlev, Denmark). This system is integrated with a CCD-chip, providing autofluorescence-spectral images (2192 × 2192 pixels; 40 μm/pixel; 32 bits/pixel) in a few seconds, requiring no sample preparation. Long-Pass (LP) filters were combined with different excitation wavelengths, providing the following excitation-emission combinations: 365/400 nm, 405/500 nm, 430/500 nm, 450/500 nm, 470/500 nm, 515/600 nm, 540/600 nm, 570/600 nm, 630/700 nm, 645/700 nm, 660/700 nm. RGB images were also captured by the same sensor, in which each individual pixel is associated with the values for red, green and blue channels.

Autofluorescences-spectral data were extracted using the VideometerLab™ software version 3.14.9 (Videometer A/S, Herlev, Denmark). It was built a mask to segment the seeds and then a normalized canonical discriminant analysis (nCDA) algorithm was applied to highlight the autofluorescence signals pixel-to-pixel. This algorithm uses 10% trimmed mean, eliminating the influence of outliers (the lowest 10% and the highest 10% of the data).

To verify whether artificial aging affects primarily the fluorophores present in the superficial regions of the seed tissues, autofluorescence images of all samples were also generated without seed coat using the same procedures described above, and they were used for illustration only.

### Germination tests

Following the imaging, seeds were distributed on a wire mesh screen suspended inside a box (11.0 cm × 11.0 cm × 3.5 cm) containing 40 mL of distilled water. The boxes were covered with lids and maintained at 25 °C for 16 h to provide a lower uptake of water. Subsequently, four repetitions of 50 seeds were placed into rolled paper towels moistened with distilled water (1: 2.5, g: mL) and kept at 25 °C with 8/16 h (day/night). The percentage of germinated seeds were based on the number of normal seedlings on the 5th and 8th days after sowing.

### Vigor tests

Four repetitions with 50 seeds per class were evaluated for electrical conductivity, SSAA, AA, and seedling emergence tests. The electrical conductivity (μS cm^−1^ g^−1^) was measured in samples previously weighed and placed in plastic containers with 75 mL of distilled water at 25 ºC for 24 h. In the aging tests, seeds were distributed over a single layer on a wire mesh screen suspended inside a box (11.0 cm × 11.0 cm × 3.5 cm) containing either 40 mL of distilled water (AA test) or 40 mL of saturated NaCl solution (i.e., 40 g of NaCl in 100 mL of water) (SSAA test), providing 98 ± 2% RH (AA test) or 76 ± 2% RH (SSAA test) at 42 °C for 72 h. The rate of normal seedlings was recorded at 5 days. The seedling emergence test was conducted in plastic boxes (32.0 cm × 28.0 cm × 10.0 cm) at room temperature (25–30 °C) for 8 days.

### Chlorophylls and carotenoids in seeds

Chlorophyll *a*, chlorophyll *b* and the total carotenoids were extracted from 5 g of seeds tissue in 10 mL of 80% acetone. After 24 h the material was filtered and centrifuged at 500×*g* for 10 min. The Chlorophylls and carotenoids were determined by the absorbance at 470, 662 and 645 nm^44,45^.

### Lipid peroxidation in seeds

Lipid peroxidation or MDA was measured according to the method described by Heath and Packer^46^. Samples with 400 mg of macerated material were mixed with 4 mL of 0.1% (w/v) trichloroacetic acid (TCA) and then centrifuged at 10,000 rpm for 15 min at 4 °C. The reaction was started by adding 250 μL of supernatant and 1 mL of 20% trichloroacetic acid + 0.5% thiobarbituric acid solution. All samples were kept in a dry bath at 95 °C for 30 min. The material was centrifuged at 10,000 rpm for 10 min, and the absorbance was read at 535 and 600 nm. The concentration of H_2_O_2_ was determined according to Alexieva et al.^47^. Approximately 400 mg of macerated material was mixed with 4 mL of 0.1% (w/v) trichloroacetic acid (TCA). The material was homogenized and centrifuged at 10,000 rpm for 15 min at 4 °C. Subsequently, 200 μL of supernatant was combined with 200 μL of 100 mM potassium phosphate buffer and 800 μL of 1 M potassium iodide solution. The absorbance was read at 390 nm.

### Lignin in the seed coat

Lignin was determined using acetyl bromide procedure as described by Fukushima and Kerley^48^. Cell wall extraction was performed with 10 mg of seed material combined with 1 mL water at 98 °C for 30 min. The samples were centrifuged for 5 min at 14,000 rpm, and then the supernatant was mixed with 1 mL ethanol and incubated at 76 °C for 30 min. Next, the samples with 1 mL of chloroform were incubated at 59 °C for 30 min. Finally, it was added 1 mL of acetone and the samples were incubated at 54 °C for 30 min. After centrifuging for 5 min at 14,000 rpm, the supernatant was removed and the pellet was dried for 45 min using a SpeedVac vacuum. The purified cell wall was used for lignin quantification using acetyl bromide.

### Total phenols in the seed coat and embryo

The total phenols in the seed coat and embryo was determined following the procedures described by Swain and Hillis^49^. The measurements were conducted in 100 mg of seed material mixed with 2.0 mL of ethanol (70%) at 70 °C water bath for 30 min. After centrifuging for 5 min at 13,000 rpm, the supernatant was combined with the Folin–Ciocalteau reagent and a sodium carbonate solution. The absorbance was measured at 725 nm.

### Photosynthesis and chlorophyll a in seedlings

Measurements were conducted on 32 pots per class (two seedlings per pot) kept at 25 °C, 8/16 h (day/night), and 50–70% RH. Light was provided by LED lamps (900 mm, 13 W) (County Ilum., São Paulo, Brazil) with a photosynthetically active radiation (PAR) of 200 μmols m^−2^ s^−1^. At 16 days, seedlings were adapted in the dark for 30 min, and then they were illuminated with high intensity amber LEDs (620 nm peak) with a saturating light intensity of 6,320 μmol m^−2^ s^−1^ in a short time interval (≈ 0.8 s). A fast chlorophyll fluorescence method based on the Kautsky induction curve was used to measure the initial fluorescence (F_0_), maximum fluorescence (F_M_), variable fluorescence (F_V_) and F_V_/F_M_^50^ using a SeedReporter™ equipment (PhenoVation B.V., Wageningen, Netherlands). A CCD-chip provided a fast screening of the chlorophyll fluorescence emission at 730 nm, generating images with a spatial dimension of 2,448 × 2,448 pixels (3.69 μm/pixel). The F_V_/F_M_ was measured based on the following formula:

$$\frac{Fv}{Fm}=\frac{Fm-F0}{Fm}.$$

Chlorophyll *a* index was measured as described by Gitelson et al.^51^ based on reflectance at 710 and 770 nm, according to the following formula:

$$Chl a index=\left(\frac{\rho 770}{\rho 710}\right)-1,$$

where *ρ* represents the spectral reflectance for each wavelength.

### Data analysis

**Figure 8:**
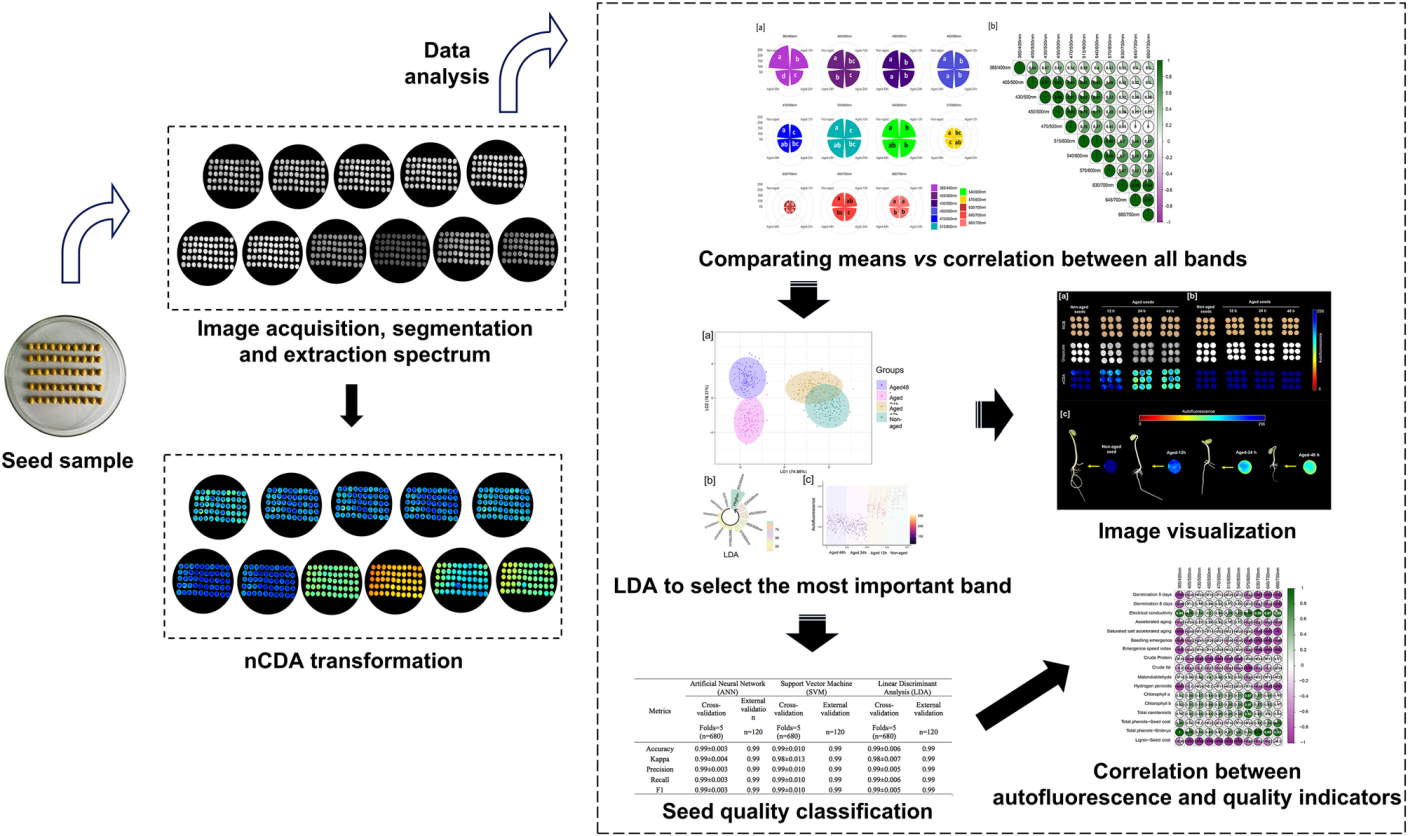
Overall flowchart of the main procedures for soybean seed quality classification based on autofluorescence-spectral imaging.

The main procedures for extracting and analyzing autofluorescence-spectral data are shown in Fig. 8. The autofluorescence values were compared by Tukey’s test (*P* < 0.05) and the Pearson’s correlation coefficient was measured to investigate the relationship among different excitation-emission combinations. A LDA was applied to rank the importance of bandwidths. Different models were developed for seed quality classification based on ANN (solver: Stochastic Gradient Descent; hidden layer sizes: two layers with 25 neurons in each; Activation function: Tanh; Learning rate: adaptive; maximum number of interactions: 5000), SVM-*l*, and LDA algorithms. Data from 680 seeds were used to train and test the models in cross-validation (K-fold = 5). An external data set with 120 seeds was used to perform external validation. The performance of the models was evaluated using five metrics—accuracy, Cohen’s Kappa coefficient, precision, recall, F1 (documentation can be found at https://scikit-learn.org/stable/modules/model_evaluation.html). The Pearson’s correlation coefficient was measured to investigate the relationship between the autofluorescence measurements and the traditional seed quality tests. All the statistical analyses were performed using R 4.0.0 software program^52^.
